# Metrology in Bioelectrical Impedance Analysis (BIA): From Measurement Science to Clinical and Research Applications

**DOI:** 10.3390/s26134017

**Published:** 2026-06-24

**Authors:** Steven Brantlov, Lars Jødal, Christian Lodberg Hvas, Søren Isidor, Charlotte Lock Rud, Jan Nielsen, Mathias Redsted, Leigh C. Ward

**Affiliations:** 1 Department of Procurement and Clinical Engineering, Aarhus University Hospital, 8200 Aarhus, Denmark; 2 Department of Nuclear Medicine, Aalborg University Hospital, 9260 Gistrup, Denmark; lajo@rn.dk; 3 Department of Hepatology and Gastroenterology, Aarhus University Hospital, 8200 Aarhus, Denmark; christian.hvas@auh.rm.dk (C.L.H.); charlotte.rud@rm.dk (C.L.R.); matred@rm.dk (M.R.); 4 Department of Clinical Medicine, Aarhus University, 8000 Aarhus, Denmark; 5 Department of Clinical Medicine, SDCA—Steno Diabetes Center Aarhus, 8200 Aarhus, Denmark; soeisi@clin.au.dk; 6 Danish Technological Institute, 8000 Aarhus, Denmark; jnn@teknologisk.dk; 7 School of Chemistry and Molecular Biosciences, The University of Queensland, Brisbane 4072, Australia; l.ward@uq.edu.au

**Keywords:** bioelectrical impedance analysis, body composition, measurement uncertainty, metrology, traceability, standardisation, clinical decision-making, impedance

## Abstract

Bioelectrical impedance analysis (BIA) is a widely used technique in clinical and research settings because it provides non-invasive estimates of body composition. However, the quality of a measurement depends on more than the perceived accuracy and precision of numbers produced by a BIA device. This review considers BIA through the lens of metrology, defined as the science of measurement. It highlights several key factors that affect measurement quality. These include accuracy, precision, calibration, standardisation, and uncertainty quantification, all of which are essential for meaningful, clinically feasible BIA measurements. Applying prediction equations generated by the device outside their intended context, poor electrode placement, or uncalibrated devices can introduce bias, whereas biological variability can complicate the interpretation of bioimpedance results. The traditional emphasis on using a reference method for validation is considered along with clinical relevance, which is argued to be an equally important benchmark for evaluating measurement utility. We also present best practices and practical guidelines for improving measurement quality, interpretation, and integration into clinical workflows. By adopting a metrological mindset in clinical practice and treating BIA with the same rigour as other diagnostic tools, its utility in areas such as fluid management, nutrition, and preventive health can be further enhanced. Trustworthy decisions depend not only on the data itself but also on how it is measured, interpreted, and used.

## 1. Introduction

Bioelectrical impedance analysis (BIA) is a non-invasive measurement method that has gained widespread use for estimating body composition, including fat mass (FM), fat-free mass (FFM), and total body water (TBW) [1,2]. The method involves passing a harmless alternating electric current through the body and measuring the body’s electrical impedance (Z), the opposition to current flow. Electrical impedance is a frequency-dependent complex quantity consisting of a real component, resistance (R), and an imaginary component, capacitive reactance (X_C_); R reflects the conductive properties of the biological tissue, and X_C_ reflects the capacitive properties [1,2].

Since the electrical conductivity of biological tissues depends on their water and electrolyte content, BIA measurements can be used to assess tissue composition. BIA measures the body’s electrical properties, which reflect the amount and distribution of conductive body fluids, as electrical current is primarily conducted through water containing dissolved electrolytes. BIA data can therefore be used to estimate body water compartments and other aspects of body composition. As an example, FFM consists primarily of muscle tissue, together with organs and the skeleton, and contains the body’s water and electrolytes. In contrast, adipose tissue (which represents most of the FM) contains much less water and is therefore largely non-conducting. Given the total body weight, BIA can be used to estimate FFM [3]. BIA devices use built-in models or prediction equations to transform the measured electrical signals (e.g., R and X_C_) into clinically relevant body composition measures, such as FM, FFM, and TBW [3].

While BIA is widely used in both research and clinical practice, its results depend fundamentally on the quality of the underlying measurements and on the assumptions underlying the transformation of electrical impedance into physiological estimates via prediction algorithms or models. From a metrological perspective, this raises important questions concerning accuracy, precision, measurement uncertainty, and suitability for the intended clinical purpose.

Based on measured raw impedance data such as R and X_C_, BIA devices commonly derive compound parameters, calculated from combinations of these impedance components, including phase angle (PhA) and impedance ratio (IR), which reflect cellular integrity and fluid distribution, respectively. In addition, some devices offer bioelectrical impedance vector analysis (BIVA), which enables users to interpret height-normalised R and X_C_ directly to assess hydration status and cellular health [4,5,6].

Common to these derived parameters is that they utilize only raw or minimally processed impedance data, rather than relying on predictive equations. In this way, the results require fewer assumptions and therefore more directly reflect the physical properties that underlie body composition and physiological processes.

BIA’s ability to assess body composition makes it a clinically useful tool across a wide range of clinical specialities, supporting the evaluation of nutritional status, guiding fluid management, and helping to track changes in health or disease [7,8]. BIA’s appeal in clinical settings is enhanced by its practicality. It is non-invasive, quick (a single measurement takes at most a few minutes), uses readily portable equipment, and is relatively inexpensive compared to imaging-based or multi-compartment reference methods, such as dual-energy X-ray absorptiometry (DXA/DEXA), computed tomography (CT), magnetic resonance imaging (MRI), or the four-compartment model [9,10]. Furthermore, the technique is safe and repeatable, involves no radiation, and causes minimal patient inconvenience [1,3,11].

Unlike most reference methods, which are typically confined to hospitals or specialised facilities [3], BIA is suitable for routine bedside use in ambulatory care, outpatient clinics, and community health settings to support assessment of fluid status and body composition [9]. In heart failure and renal care, BIA-derived fluid parameters, such as extracellular water (ECW) and BIVA, have been explored to guide diuresis and detect fluid overload, underscoring their value in assessing fluid distribution alongside traditional measures [12].

However, conventional wrist-to-ankle whole-body BIA is relatively insensitive to truncal fluid accumulation because limb impedance contributes more strongly to the measured signal primarily due to their smaller cross-sectional area. Consequently, fluid-related parameters in conditions such as heart failure should be interpreted cautiously and in conjunction with clinical assessment, longitudinal trends, and, where available, segmental or complementary measurement approaches [13].

Despite these advantages, the interpretation of BIA data must be approached with caution and scientific rigour, particularly from a measurement and metrological standpoint.

By its nature, BIA does not directly measure body composition; instead, it measures the electrical properties of tissues from which body composition is estimated [1,3]. The accuracy of these estimates depends on several factors, including measurement conditions and the validity of the predictive models employed [1].

In practice, clinicians and researchers have encountered variability in BIA results due to differences in measurement protocols, including electrode placement, patient hydration status, recent food or exercise intake, and device-specific predictive algorithms [7,14]. In addition, confusion about terminology and concepts, such as metrological principles like accuracy and precision, as well as measurement uncertainty, can lead to miscommunication and misinterpretation of results, especially when BIA output is treated as exact values. Metrology, the science of measurement, provides the framework for ensuring that such measurements are trustworthy and clinically meaningful [15,16]. These challenges are not unique to BIA but mirror those of other medical measurement methods, in which fundamental metrological principles, such as the distinction between accuracy and precision, proper consideration of measurement uncertainty, traceability, and appropriateness for intended clinical purposes, are often overlooked [15,16].

Inconsistency in measurement definitions and a lack of standardisation can hinder both clinical communication and the proper use of devices [15,17]. While clinical chemistry has long emphasised quality control and standardisation, BIA remains poorly standardised despite long-standing recommendations in this regard [1,11]. A persistent lack of methodological consistency in BIA studies, particularly in paediatric populations, has been noted, emphasising the need for device- and population-specific protocols [1,18,19].

In the context of BIA, a better understanding of metrological concepts and an appreciation of their importance are essential to ensure high-quality measurements and to fully realise the technique’s clinical potential and acceptance.

This review examines BIA from a metrological perspective, focusing on how BIA measurements are translated into clinically meaningful information using fundamental measurement principles. It analyses how uncertainty, arising from patient-related, environmental, technical, and methodological sources, propagates through the measurement system and affects the trustworthiness of derived parameters. Emphasis is placed on standardisation and traceability to the International System of Units (SI), as well as on best practices for patient preparation, electrode placement, and device calibration. The validity of BIA-derived outcomes is critically assessed based on technical performance, biological plausibility, and clinical utility. Metrological reasoning is proposed as a foundation for data quality management, enabling interpretation of measurement errors in the context of clinical decision-making involving BIA.

Therefore, the review offers practical implementation tools, including a structured checklist and example uncertainty budgets, to support users throughout the measurement-to-interpretation process.

Finally, this review is structured to bridge three interconnected levels: (1) general metrological principles relevant to clinical measurements, (2) BIA-specific measurement and modelling challenges, and (3) interpretation of BIA-derived parameters in clinical decision-making.

## 2. BIA as a Measurement System

BIA involves various measurement techniques and device types, each with specific capabilities, limitations, and use-case scenarios [1]. Understanding this variability is essential in any metrological evaluation of a measurement system, such as BIA, because the choice of technique directly affects overall measurement performance and, hence, clinical utility.

BIA techniques range from simple, single-frequency devices to advanced instruments that employ full-spectrum impedance analysis [1,20]. An overview of the available BIA techniques and their technical and clinical characteristics is provided in Table 1.

Single-frequency BIA (SF-BIA) is the most basic implementation, typically utilising alternating current (AC) with a fixed frequency of 50 kHz, to estimate TBW [1,3,20].

It is valued for its simplicity and affordability, but cannot reliably differentiate extracellular and intracellular compartments. Multiple-frequency BIA (MF-BIA) may improve the estimation of fluid compartments compared with single-frequency BIA by incorporating multiple frequency measurements, typically three to six, within the range 5–500 kHz, to discriminate more effectively between ECW and intracellular water (ICW) compartments [1,3,20]. Some devices use only two frequencies (e.g., 1 and 100 kHz) and are referred to as dual-frequency BIA (DF-BIA), which is considered here a special case of MF-BIA. At the top of the technological spectrum is bioimpedance spectroscopy (BIS), which measures impedance over a wide frequency range, typically using 50 to 256 discrete frequencies between 2 and 1000 kHz [1,3,20]. In addition, BIS typically uses biophysical modelling of impedance parameters (R and X_C_), commonly based on the Cole model, to characterise tissue properties (e.g., cell membrane capacitance) and to estimate fluid distribution between extracellular and intracellular compartments (ECW and ICW) [20] using Hanai mixture theory [21] rather than empirically derived prediction equations as in SF-BIA and MF-BIA.

However, BIS does not eliminate modelling assumptions, as estimation of body fluid compartments still depends on assumptions related to tissue resistivity coefficients implicit in mixture theory [20]. The practical advantage of BIS is therefore not the absence of assumptions, but a greater reliance on frequency-dependent impedance measurements and biophysical modelling compared with purely empirical regression-based approaches.

Furthermore, the underlying assumptions of Hanai mixture theory may be less valid under altered body geometry or fluid distribution, such as in severe obesity, potentially increasing model-dependent uncertainty in ECW/ICW estimates [3,20].

Beyond the frequency spectrum used, BIA devices also differ in electrode configuration and measurement sites. Some devices use fixed electrodes in hand-held, stand-on, or combined hand-to-foot systems. Others use adhesive surface electrodes, typically silver/silver chloride (Ag/AgCl), as used for ECG measurements, and connect them by wires, as in lead-type BIA devices [22]. These differences influence the path of the electrical current through the body, thereby determining which body regions contribute to the measurement and the ability to distinguish impedance within specific segments, an important consideration when interpreting BIA results in clinical and research settings. They also determine whether the device is suitable for use in a standing, supine, or sitting posture. Similarly, measurement sites can be whole-body, segmental, or focal, depending on the clinical purpose [1,13].

Another key difference is between body composition estimates derived from prediction equations and bioimpedance-derived parameters calculated directly from measured R and X_C_. Prediction equations provide easily interpretable clinically relevant values, such as FFM (kg) or TBW (litres). However, they are sensitive to population-specific variables (e.g., sex, age, ethnicity, disease) and to modelling assumptions [1]. In contrast, bioimpedance-derived parameters such as PhA, which do not rely on body-composition prediction models, may offer more direct physiological insight and be less dependent on population-specific assumptions.

Similarly, impedance ratio should not be interpreted as a direct surrogate to BIS-derived ECW/TBW estimates, as these parameters are based on different modelling approaches and physiological assumptions. Rather, impedance ratio may provide complementary information related to frequency-dependent impedance behaviour and fluid distribution.

Common bioimpedance parameters across BIA techniques are summarised in Table 2.

The disadvantage of raw impedance data and derived parameters is that they do not yield absolute compartment values and are therefore less intuitive to interpret in routine clinical practice. However, in research and in precision medicine contexts, where treatment is tailored to the individual patient, such parameters may be particularly valuable.

**Figure 1:**
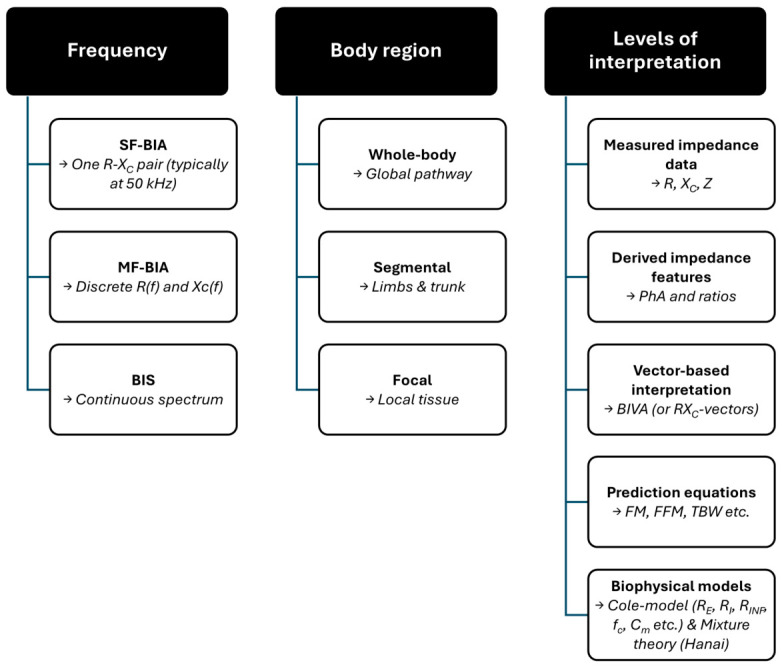
Conceptual framework for BIA as three independent dimensions. The figure illustrates three dimensions of BIA: frequency, body region, and levels of interpretation. Frequency and body region define the measured impedance data, which may be used directly or further processed into derived features, vector-based interpretations (e.g., BIVA), prediction equations, or biophysical models. The interpretative levels reflect increasing abstraction from measured data rather than a strict hierarchy.

For these reasons, BIA is not a single technology but a family of techniques, each with different levels of complexity, accuracy, and clinical relevance. These relationships are summarised conceptually in Figure 1. These methodological distinctions must be considered when assessing the metrological properties of BIA techniques, interpreting results, and comparing data across devices or studies.

## 3. BIA Device Performance and Clinical Measurement Quality

Understanding and evaluating the quality of BIA measurements requires attention to both the device’s technical performance and the clinically relevant measurement properties. This section outlines key metrological concepts and clinical performance indicators that influence the robustness of BIA results. Table 3 summarises all terms introduced in the text and provides an overview of commonly used BIA parameters, grouped by their relevance to accuracy, precision, agreement, and clinical utility.

### 3.1. Instrument Performance and Signal Properties

Correct bioimpedance measurements depend on the technical performance of the BIA device and on the appropriate measurement protocol. Key technical characteristics of a measurement device influence the quality and consistency of measurements, which, in turn, affect their clinical usefulness [1,23].

The technical performance of a BIA device is constrained by its manufacturing specifications:

Frequency response. In BIA instruments, the practical measurement range is typically limited by safety considerations and signal-to-noise constraints to approximately 1–1000 kHz [3,20]. Within this range, lower frequencies (e.g., 1–5 kHz) predominantly reflect ECW, whereas higher frequencies (50–1000 kHz) reflect TBW [1,3,20].

Measurement range. The range of a measurement device is the span between the minimum and maximum values it can measure accurately [17,23]. In BIA, the measurement range depends on the device design and intended application, but commercial devices are generally designed to accommodate the impedance values encountered in human subjects [20,24]. Whole-body impedance measured at 50 kHz varies substantially among individuals and is influenced by factors such as body size, body composition, sex, age, and hydration status [3,20]. Measurements performed outside a device’s specified measurement range should be interpreted with caution, as measurement performance cannot be assumed to remain within the validated specifications [17,23].

Measurement resolution. In BIA devices, resolution refers to the device’s ability to detect small differences in electrical properties, such as impedance [23]. Although it contributes to measurement uncertainty, resolution is usually much smaller than uncertainty arising from other sources, including device accuracy, noise, calibration, and biological variability [17,23]. Clinically, higher resolution allows a device to detect smaller changes. For example, a BIA device with a resolution of 0.01 Ω can detect more subtle changes in fluid status or tissue composition than one with a resolution of 0.1 Ω, provided the changes are large enough to be distinguished from normal measurement variability [23].

Linearity of response. In BIA, linearity ensures that changes in impedance reflect proportional changes in physiological properties such as fluid volume or tissue composition [3]. Poor linearity introduces systematic errors that distort derived values such as FFM or PhA.

Electronic accuracy. Device specifications typically state the measurable impedance range and the associated measurement accuracy. If, for example, a device accuracy of ±1% is assumed, a whole-body impedance value of 500 ohm (Ω) would correspond to approximately 495–505 Ω. Measurement accuracy is limited by the device specifications. Electronic (device) accuracy contributes to but should not be confused with the accuracy of clinical outcomes derived from impedance measurements.

Other characteristics of electronic measurement devices, such as frequency response and signal fidelity [23], are not commonly provided for BIA devices, but appropriate manufacturing quality is assumed.

Device specifications should be routinely verified with reference standards (electronic circuits of known value) with periodic calibration to establish metrological traceability through independent calibration or validation by accredited service providers.

Beyond periodic calibration, modern biomedical devices increasingly incorporate built-in functions that continuously monitor measurement quality and device performance during routine use [25]. Such functions can help detect measurement deviations, support traceability to calibrated reference standards, and improve confidence in the reliability of reported results.

### 3.2. Metrological Concepts and the BIA Measurement-to-Decision Chain

Metrology offers a framework linking measurement to clinical decision-making. Applied to BIA and grounded in medical instrumentation principles [23], it defines a five-stage measurement-to-decision chain:

1. Defining the measurand and selecting the measurement method.
2. Acquiring impedance data under standardised conditions.
3. Processing the data into clinically relevant parameters using device-specific algorithms.
4. Interpreting results in relation to reference values, trends, or clinical context.
5. Applying the results to inform clinical decisions such as monitoring, risk assessment, or therapy adjustment.

**Figure 2:**
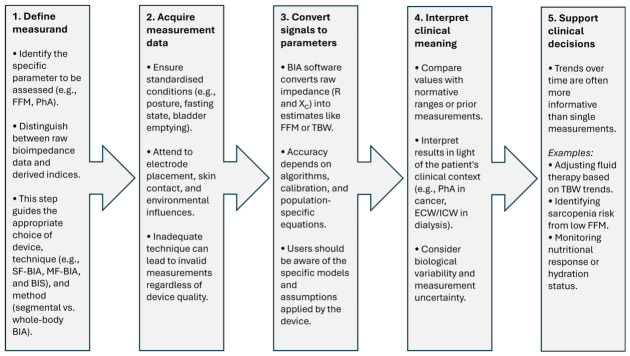
BIA measurement-to-decision chain. This figure visualises the five key stages from defining the measurand to making clinical decisions, each of which can introduce uncertainty and should be managed carefully within a metrological framework.

Each stage introduces potential sources of error and uncertainty that may propagate through the chain if not properly managed. Figure 2 illustrates this process and highlights how measurement quality at each stage influences the overall reliability and clinical utility of BIA.

### 3.3. Measurands and Measurement Principles in BIA

The measurand is defined as “the quantity intended to be measured” [17]. In BIA, this quantity is the electrical impedance of the body or a defined body segment, measured under specified conditions. Impedance is commonly measured at a single frequency of 50 kHz in SF-BIA or across a range of frequencies using MF-BIA or BIS [1,3,20].

In standard whole-body BIA, the measurand is the complex impedance (Z) measured between two current-injecting electrodes and two voltage-sensing electrodes, typically positioned from wrist to ankle in a tetrapolar (four-electrode) configuration [3]. The measured impedance reflects the body’s conductive (resistive) and capacitive (reactive) properties, which are influenced by tissue composition, including water distribution, electrolytes, and cell membranes.

In routine clinical use, many BIA devices do not display the underlying impedance measurements; instead, they automatically transform the measured impedance into body composition estimates using built-in algorithms. Consequently, two related measurands can be distinguished: a direct measurand, the measured electrical impedance (e.g., R and X_C_), and an indirect measurand, the predicted body composition parameter (e.g., FFM or TBW) derived from impedance data using a device-specific model [26].

Limited access to raw impedance data in some commercial BIA devices may represent a practical barrier to fully transparent metrological evaluation and interpretation. Greater availability of such data and clearer reporting of device-specific prediction equations would improve reproducibility, comparability, and clinical interpretation. Nevertheless, core metrological principles such as calibration, standardisation, traceability, uncertainty evaluation, and consistent longitudinal measurements remain important even when only derived parameters are available to the user.

The measurement technique in BIA is based on a physical principle derived from Ohm’s law, voltage (U or V) = resistance (R) × current (I), as applied to the human body [3]. Interpretation of impedance data may rely on conductive volume models, in which the body or body segments are approximated as cylindrical conductors, and/or on empirically derived prediction equations based on regression analysis against reference methods [3,20]. More advanced approaches, particularly in BIS, further incorporate frequency-dependent electrical modelling and mixture theories, such as Cole and Hanai models, to estimate body fluid compartments [3,20].

A measurement result is not the true value of the measurand, which cannot be known exactly, but an estimate accompanied by measurement uncertainty [27]. While this principle applies to all medical measurements, it is particularly important in BIA, where clinically used outputs are derived rather than directly measured quantities. Therefore, from the outset, it should be acknowledged that BIA body composition estimates are model-dependent measurements that carry uncertainty arising from both the electrical measurement itself and the biological assumptions embedded in the device’s prediction model [27].

### 3.4. From Technical Accuracy to Clinical Validity

Accuracy, precision, agreement, and uncertainty are key concepts for evaluating the performance of BIA measurements. Accuracy describes the closeness of agreement between a measured value and a reference value, whereas precision describes the consistency of repeated measurements under specified conditions [17].

Agreement between BIA and reference methods is commonly assessed using Bland–Altman analysis, which evaluates both the magnitude and distribution of differences across the measurement range [28,29]. Importantly, good agreement with a reference method does not necessarily imply that a measurement is suitable for all clinical purposes.

Precision underlies repeatability and reproducibility, both of which are essential for longitudinal monitoring. In BIA, random variability may be quantified using the technical error of measurement (TEM), which assesses variability arising from repeated measurements by the same or different operators [30].

Measurement uncertainty provides an estimate of the range within which the true value is expected to lie and is particularly important when interpreting small changes in bioimpedance parameters over time [27]. Because BIA outputs are influenced by both measurement variability and model assumptions, uncertainty should be considered when evaluating clinically meaningful change.

Finally, validity refers to the extent to which a measurement is appropriate and meaningful for its intended clinical purpose, population, and context of use. A method may demonstrate good technical performance yet remain clinically inappropriate if applied outside its intended setting. Conversely, a method with imperfect agreement may still provide clinically useful information when interpreted within an appropriate clinical framework.

### 3.5. Agreement and Correlation Metrics

Assessing the relationship between measurements, whether across methods, time points, or operators, is crucial for determining both consistency and validity. Various statistical tools are employed to quantify agreement and correlation, depending on the nature of the data and the purpose of the comparison.

A good starting point for comparing two measurement methods or two devices is to perform paired measurements with both methods. Plotting one method against the other can provide a clear visual impression of the relationship (or lack thereof) between the two methods. Computation of the concordance correlation coefficient *r* (Lin’s correlation coefficient) can supplement the visual impression. If the differences between method X and method Y are normally distributed, then *r*^2^ reflects the fraction of the variance in Y that can be explained by the variance of X, e.g., *r*^2^ = 0.8 = 80% means that only 20% of the variance of Y is unrelated to X.

Correlation is not, however, a measure of agreement, which can be evaluated using Limits of agreement (Bland–Altman) plots. For each measurement, the difference between the two methods is plotted as a function of the mean of the two methods. This approach puts the two methods on equal footing, avoiding the need to designate one method as the reference method [28].

To evaluate consistency across repeated measurements or between operators, the intraclass correlation coefficient (ICC), similar to Lin’s concordance correlation coefficient, is generally considered appropriate [31]. Unlike method-comparison metrics, the ICC is designed to assess intra- and inter-operator reliability by accounting for both correlation and agreement within clustered data, such as repeated measurements obtained from the same subject. A larger ICC indicates greater measurement consistency. While values <0.5, 0.5–0.75, 0.75–0.9, and >0.9 are often used to describe poor, moderate, good, and excellent reliability, respectively, such thresholds are context-dependent and should not be interpreted as absolute criteria [31].

### 3.6. From Measurement to Clinical Relevance

In the application of body composition assessments, such as BIA, measurement metrics must be not only reliable but also clinically meaningful. Several concepts help bridge the gap between technical accuracy and clinical decision-making.

Metrological traceability refers to the formal association between a measurement and a reference standard, typically established through calibration procedures [17]. In BIA, traceability ensures that outputs are aligned with known quantities or reference methods (e.g., DXA or isotope dilution), thereby supporting the validity and comparability of results across devices, studies, and clinical settings [7]. This traceability provides the foundation for meaningful assessment of measurement error and clinical interpretation.

In clinical practice, BIA is often interpreted alongside complementary assessment modalities such as DXA, CT, MRI, ultrasound, biochemical markers, and clinical examination. Integrating information from multiple assessment methods may provide a more comprehensive understanding of body composition, physiological status, and health than any single method alone. Such approaches may also help clinicians recognize the limitations and assumptions associated with individual methods and prediction models [1,2].

In severe clinical conditions, such as critical illness with large fluid shifts, oedema, inflammation, or poor tissue perfusion, the relationship between impedance measurements and the patient’s actual physiological state may become less predictable [1,3,7]. Even raw impedance data may therefore be difficult to interpret in isolation. Although the use of such data may mitigate some limitations associated with violations of the assumptions underlying prediction equations, interpretation should remain guided by the broader clinical context and other relevant physiological and clinical information [1].

Bias, or systematic error, reflects a consistent deviation from the reference value. Systematic errors can often be identified (e.g., bias as assessed by Bland–Altman (LoA) analysis) and corrected through calibration or adjustment, improving the overall accuracy of the measurement system.

In contrast, random error refers to unpredictable variation that occurs by chance and affects the consistency of repeated measurements. These cannot be corrected directly and are instead described statistically, using metrics such as standard deviation (SD) or coefficient of variation (CV), while confidence intervals (CI) are used to express the uncertainty of estimated parameters.

In practice, both systematic and random errors may arise at multiple stages of the BIA measurement chain, including signal acquisition, electrode–skin interface, device hardware, and data modelling. These error components are generally treated as contributing additively (in terms of variance) and propagate to the final output values, thereby contributing to the combined measurement uncertainty [27]. To determine whether a change in measurement exceeds the threshold of error, the minimal detectable change (MDC) is typically used [32]. This is the smallest measurable change that can be interpreted as real, i.e., beyond random variation, and is calculated as follows:

$$MDC=z\cdot SEM\cdot \sqrt{2}$$

where

$$SEM=SD\cdot \sqrt{1-ICC}$$

Here, *z* denotes the standard normal quantile corresponding to the desired confidence level, e.g., 1.96 for a 95% confidence level.

Lastly, the minimal clinically important difference (MCID) is the smallest change in a measurement that is meaningful from a clinical perspective, i.e., a change that patients or clinicians consider beneficial. While the MDC is derived from statistical properties, the MCID reflects clinical relevance [33]. Unlike the MDC, the MCID has no fixed formula and depends on context. It is often based on clinical judgment, comparisons to other measures, or patient feedback.

The number needed to treat (NNT) is an indicator of clinical effectiveness [34]. It represents how many patients need to be treated for one person to benefit from the intervention and avoid an adverse outcome, and is calculated as:

$$NNT=\;\frac{1}{Risk\;difference}=\frac{1}{\pi_{1}-\pi_{0}}$$

The risk difference represents the difference in the proportion of patients who experience an adverse outcome between the treatment group (*π*_1_) and the control group (*π*_0_). A larger risk difference indicates a greater treatment effect, resulting in a lower NNT and greater clinical effectiveness. An example of how NNT can operationalise the clinical impact of measurement-guided intervention is provided by a RCT of non-invasive lung impedance–guided management in chronic heart failure, in which a significant reduction in acute heart failure hospitalisations over 12 months corresponded to an NNT of approximately 1.4 based on observed event rates [35].

### 3.7. Modelling and Assumptions Underlying BIA Outputs

Once impedance data (e.g., R and X_C_) are measured, BIA devices convert these electrical measurements into clinically meaningful body composition parameters using built-in prediction equations or biophysical models [1,3,7]. The specific outputs, therefore, depend on the device type and the modelling approach applied. To interpret BIA-derived parameters appropriately, it is essential to understand the assumptions underlying these models.

In SF-BIA and MF-BIA, body fluid compartments are typically estimated using empirically derived prediction equations [1,3,7]. A classic example is the estimation of TBW based on the impedance index (height^2^/impedance), which assumes the body behaves as a cylindrical conductor and requires empirically derived coefficients [3]. These coefficients are obtained under defined conditions, such as euhydration, a fixed body position (e.g., supine or standing), and specific demographic characteristics including age, sex, body type, or ethnicity. When the measured individual or clinical scenario deviates from these conditions, additional measurement uncertainty is introduced because the prediction model may no longer perform as intended [1].

Although these simplified cylindrical assumptions and largely ignore anisotropy of tissues have enabled practical clinical implementation of BIA, they may limit physiological specificity in heterogeneous tissues and altered fluid states. More advanced approaches based on spatial impedance reconstruction and tomographic modelling have therefore been proposed to improve localisation and physiological interpretation of impedance data and to reduce limitations associated with conventional regression-based and cylindrical body models [36]. However, such approaches currently remain primarily research-oriented and are not yet part of routine clinical BIA/BIS practice.

In contrast, BIS estimates body water compartments by modelling frequency-dependent impedance using the Cole model to derive resistance at zero (R_0_) and infinite frequency (R_∞_), which are subsequently converted into estimates of TBW and ECW using Hanai’s mixture theory (a biophysical model relating electrical resistance to fluid volumes) [20,37,38]. This approach reduces reliance on population-specific prediction equations used in SF-BIA and MF-BIA [1,20]. However, BIS is not free from empirical assumptions, as it still requires calibration against reference methods to derive tissue resistivity coefficients for ECW and ICW [20,38,39].

In addition, biological relationships underlying BIA measurements may not behave linearly across all physiological and clinical conditions. Changes in hydration, tissue composition, inflammation, obesity, and fluid distribution may alter conductive pathways and impedance behaviour in ways that are not fully captured by simplified regression-based models. Such physiological complexity may therefore contribute to model-dependent uncertainty and reduced generalisability across populations and disease states [1,3,20,40].

Clinical conditions such as oedema, cachexia, severe obesity, and inflammation may further challenge conventional BIA modelling assumptions because fluid distribution, tissue conductivity, and body geometry may differ substantially from reference populations used to derive prediction equations. In such situations, interpretation may rely more heavily on raw impedance parameters (e.g., PhA and BIVA) and longitudinal trends rather than absolute body composition estimates alone.

After data processing, the device software typically displays results such as “FM = 20 kg, FFM = 55 kg”, typically with limited numerical precision (e.g., rounded to the nearest kilogram or the first decimal place), and may also include normative ranges.

It is tempting for users to treat these numbers as direct measurements, but they are inferences derived from multiple layers of measurement and modelling. Each value has an implicit uncertainty that users should be aware of, even if the device does not display this.

Although a person’s FFM may be displayed as a single rounded value (e.g., 54 kg), this represents an estimate of the true value rather than an exact measurement; the true FFM is expected to lie within an uncertainty interval determined by combined physiological, technical, and modelling uncertainties.

Emerging data-driven and artificial intelligence (AI)-based or machine learning approaches may further expand the interpretation of bioimpedance data [41,42,43]. However, such models remain dependent on the quality and standardisation of the underlying measurements and may introduce additional uncertainty related to model training, population selection, and algorithmic assumptions [41,42,43]. From a metrological perspective, AI-derived outputs should therefore be evaluated with the same emphasis on validation, uncertainty assessment, transparency, and clinical applicability as conventional prediction models [41,42,43].

## 4. Standardisation and Quality Control

### 4.1. Importance of Standardisation

BIA is highly sensitive to methodological variation. Accordingly, standardisation, defined here as the consistent use of measurement procedures and reference conditions, is essential to ensure valid comparisons and reduce confounding physiological variability, consistent with general metrological principles [17]. Expert groups have repeatedly emphasised that the lack of standardisation and quality control is a key barrier to clinical adoption of BIA in both adult and paediatric populations [7,11,14,18,19].

Standardisation is achieved through structured protocols (standard operating procedures, SOPs) that govern patient preparation, measurement timing, body position, electrode placement, and device maintenance. While adult protocols have been available for some years [7,11,44], recent efforts have underscored the need for population-specific adaptations for paediatric populations [14,18,45,46].

### 4.2. Controlling Physiological Variability

Recommendations generally address several factors that influence BIA measurements, including overnight fasting (at least a few hours without food or drink), avoidance of strenuous exercise and alcohol intake, and other hydration-related factors prior to measurement [7,11,14]. Voiding before measurement is recommended but appears to have only a minor effect on whole-body impedance (≈1%) [44] and may not always be feasible in clinical practice [14]. While such standardisation is important to reduce variability and improve measurement precision, and therefore is essential in controlled research settings to minimise noise and increase statistical power, it may not always be feasible in routine clinical settings, where less controlled conditions must be considered.

Furthermore, the person being measured should either lie supine (after a few minutes’ rest to stabilise fluids) or stand upright, depending on the design of the device. Most importantly, the same position should be used each time. For supine measurements, keep the legs apart (not touching) and the arms away from the trunk to avoid electrical interference during current flow through the body. For stand-on BIA scale-type devices, the feet must be correctly positioned on the footplate electrodes, and the hands should grip the hand electrodes, if present.

Consistency in electrode placement, particularly important for lead-type BIA devices, is ensured by using anatomical landmarks; for example, the wrist electrode is placed at the level of the ulnar styloid processes, and the ankle electrode at the medial malleoli when using gel electrodes. If repeated measurements are to be obtained on the same subject over time, marking the electrode sites can help maintain repeatability. The environment should be controlled by measuring in a room with a comfortable, stable temperature, i.e., not immediately after being in a very hot or cold environment, and ideally at a consistent time of day. To be confident that a change is real, strict adherence to measurement protocols is essential. Normal day-to-day physiological variability is inherent and must be considered when interpreting serial measurements to distinguish true clinical change from normal biological variation. For example, changes in TBW of roughly 0.5 L or in FFM of about 0.5–1.0 kg (approximately 1–2% of typical adult values) may be required before a change exceeds normal measurement variability and can be considered potentially clinically meaningful. However, the exact threshold depends on the precision of the device used [47].

To support broader acceptance of BIA for routine clinical use, there is a need for consensus-based checklists and formal accreditation of testing procedures, such as standardisation procedures known in clinical laboratories [1].

Practical consequences of physiological variation are significant. Hydration status strongly affects BIA readings: dehydration increases R, which is approximately proportional to 1/TBW [3,48], potentially leading to underestimation of TBW and FFM, whereas fluid overload lowers R and may result in overestimation of TBW and FFM. For example, if a person’s TBW is measured at 40 L, the estimated FFM depends on how much water is assumed to be in that tissue. Using the following relationship:

$$FFM=\frac{TBW}{0.732}$$

As applied in many prediction equations [3], a TBW of 40 L corresponds to an FFM of 55 kg.

With essentially no water in the FFM, the relationship corresponds to assuming a constant FFM hydration of 73%. If, however, the actual hydration of FFM is higher, say, 76%, the FFM estimate decreases to 40/0.76 = 53 kg, indicating a relative expansion of body water, which may be due to disease-related fluid shifts, acute fluid intake, physiological variation, or methodological factors, and illustrating the limitations of constant-hydration assumptions.

Physiological models suggest a mean hydration fraction of ≈0.73 with individual variation typically falling within a narrow range (≈0.69–0.77) in healthy adults [49,50,51].

Notably, however, the hydration fraction is much higher in newborns and infants; tables of normative hydration values for these populations are available [50,52,53].

The posture during measurement affects fluid distribution in the body, which, in turn, influences electrical resistance. When lying down (supine), gravity no longer causes fluid to pool in the legs, and body fluids redistribute centrally toward the trunk, where the body has a larger conducting volume. As a result, the electrical measurement is less dominated by the long, narrow limb segments, thereby effectively shortening the conduction path length (L) and increasing the effective cross-sectional area (A), thereby lowering R. In contrast, when standing, gravity promotes peripheral fluid pooling in the legs, increasing the effective path length and reducing the cross-sectional area for conduction in the torso, thereby increasing R.

These postural effects follow the relationship R∝L/A, where R increases with conductor length and decreases with cross-sectional area [3], and have been demonstrated experimentally in posture-dependent bioimpedance studies [54]. Thus, impedance data are not directly interchangeable between postures or between measurements with different electrode positions, e.g., gel electrodes at the styloid processes & malleoli versus hand-held & stand-on electrodes.

Table 4 summarises how common measurement conditions, such as posture, hydration, and recent activity, can systematically affect BIA parameters. These variations highlight the need to standardise measurement, timing and positioning (e.g., in the morning, post-voiding, before meals, or before exercise). When standardised measurement protocols are followed, modern BIA devices can achieve test–retest variability of less than 1% [47], thereby facilitating the reliable detection and interpretation of small physiological changes.

### 4.3. Biological and Technical Uncertainty Sources

No measurement is perfect, and uncertainty arises from several interacting sources. In BIA, these can be categorised into: (1) participant-related physiological variability, (2) environmental or procedural variation, (3) instrumentation, and (4) algorithmic modelling.

Biological variation reflects day-to-day within-subject fluctuations around an individual’s homeostatic “set point,” driven by physiological regulation and factors such as diet, physical activity, and age. These are genuine physiological changes, distinct from analytical (measurement) variation [55]. For the most precise instruments to detect such changes, it is necessary to distinguish these from technical error; repeated measurements over several days are needed, ideally analysed using statistical techniques that separate biological and technical variability [47].

Procedural and environmental variation, such as recent meals, time of day, or room temperature, introduces additional uncertainty. These factors can amplify or mask physiological changes, making protocol consistency essential to avoid misinterpretation.

Instrumentation can also contribute to measurement error. In four-electrode (tetrapolar) bioimpedance measurements, the influence of electrode and lead resistance on the measured impedance is largely reduced by the measurement principle and is typically small under normal measurement conditions. In contrast, in two-electrode systems, electrode and lead resistances are included in the measured impedance. Under non-ideal conditions, such as increased lead resistance due to cable properties or degraded connections, small technical contributions to the measured resistance may nevertheless occur [56]. A metrological study [57] investigated the influence of technical factors, including external lead cables, on systematic errors in BIA measurements and reported that cables, electrodes, and contact gel had a negligible effect on the overall measurement chain, with measurement uncertainties below 0.35 Ω (68% confidence interval, CI) for both R and X_C_. These findings support that, when used as intended, cables and electrodes are not a major source of measurement error, although calibration and validation under real-world conditions remain essential.

Devices with integrated stainless-steel electrodes (e.g., foot-to-foot or hand-to-hand devices) avoid separate lead wires and adhesive electrodes, but remain subject to other configuration-related sources of variability.

Together, these biological and technical factors underscore the need for well-controlled protocols, standardised posture, timing, and ambient conditions, to reduce intra-individual variability and improve the reliability of BIA in clinical or research settings.

Beyond measurement, BIA-derived physiological parameters are influenced by the assumptions embedded in the algorithms used to calculate them. estimates. These assumptions include fixed values for FFM hydration (≈73.2%), constant electrolyte distributions, and population-averaged tissue densities. In addition, prediction equations rely on measured inputs such as height and body weight, so any measurement error in these variables propagates through the equations and adds uncertainty to the final estimates [38]. Such assumptions may introduce systematic errors, particularly when applied to populations with altered body composition or fluid balance, such as very lean or muscular individuals and patients with fluid overload (e.g., oedema), where FFM hydration deviates from population averages [50]. As illustrated above, FFM estimates depend directly on the assumed hydration constant. Even modest deviations in true hydration (e.g., 72–76%) can change FFM by approximately ±2 kg (±5%), a difference that is clinically meaningful relative to commonly used cut-offs and may increase the risk of misclassification. Inter-individual variation in FFM hydration has been documented in healthy adults, supporting the view that the commonly assumed hydration constant represents an approximation rather than an exact physiological value [51].

Moreover, raw impedance data (e.g., R and X_C_) are also affected. R decreases with fluid overload. As ECW increases, X_C_ may decrease because the electrical current flows more readily through the fluid and is less influenced by cell membranes. This primarily reflects fluid redistribution rather than a true loss of FFM or cellular integrity. Such systematic shifts in measured bioimpedance parameters, including reductions in X_C_ and changes in R driven by altered ECW distribution, can be misinterpreted as changes in tissue properties or cellular health, even when body composition estimates are not calculated. These effects are not random but arise predictably under specific physiological conditions. Although they cannot be eliminated, they can be mitigated using the following interpretation strategies:

- Use MF-BIA or BIS to estimate ECW and ICW separately [3];
- Monitor ratios like ECW/TBW (>0.40 may suggest overhydration) [58,59];
- Use PhA, derived from R and X_C_, as a composite indicator influenced by both cellular properties and hydration status [5];
- Avoid assuming a fixed FFM hydration of ≈73.2% when interpreting bioimpedance data in populations with altered fluid balance [9];
- Use both the measured impedance values (e.g., R and X_C_) and the modelled body composition estimates (e.g., FFM, FM) when interpreting results [60];
- Interpret measurements alongside clinical findings and relevant patient information (e.g., hydration status, disease state, recent treatments) [7,14].

Another frequently overlooked issue is the inherent uncertainty of reference methods to provide “true” values, such as DXA or dilutional analysis [61]. DXA, for example, exhibits measurable precision error in fat percentage (e.g., ≈0.8–3% CV on repeat scans) [62,63] and is sensitive to variations in soft-tissue composition and hydration, which affects accuracy and calibration interpretation [64,65].

In whole-body BIA, R dominates the impedance signal. In healthy adults measured at 50 kHz, R values (approximately 450–600 Ω) are substantially larger than X_C_ (approximately 40–70 Ω or ~10% of R), indicating that R accounts for most of the total impedance. Consequently, estimates of TBW are driven primarily by R, as reflected in the height^2^/R relationship used in prediction models [3,66,67,68].

These outputs are subject to combined uncertainty arising from measurement repeatability, physiological variability (e.g., hydration status and posture), and modelling assumptions. As a result, reporting values with excessive numerical precision may imply a level of accuracy not supported by either device performance or normal biological variation, and small numerical differences may be misinterpreted as clinically meaningful. Numerical reporting should therefore reflect the device’s effective resolution and expected physiological variability, rather than the software’s default decimal output. Careful consideration of these modelling- and representation-related uncertainties is essential for robust interpretation of BIA results in both clinical and research settings.

### 4.4. Calibration and Traceability

All BIA devices intended for clinical use or placed on the market as medical devices should be regarded as measurement instruments in the metrological sense. As such, their performance should be regularly verified through appropriate calibration and validation procedures.

Most manufacturers supply test components (e.g., fixed resistors or resistor–capacitor (RC) circuits) that simulate known body-impedance values, enabling device measurements to be checked across different frequencies. These test components are intended for routine performance verification and do not replace proper calibration. However, while simple resistors are sufficient to validate R, they do not enable meaningful assessment of X_C_ or the PhA. For this purpose, RC or more complex resistor–resistor–capacitor (RRC) circuits are required to introduce a capacitive component that more closely represents tissue-like electrical behaviour [69].

When such circuits are connected in place of the patient, a properly functioning device would be expected to return values close to the known reference. For example, if a device consistently reports a 500 Ω reference resistor as approximately 505 Ω, this indicates a systematic deviation (bias) from the reference value. In practice, such bias might prompt calibration verification or recalibration. Even small systematic deviations may influence measurement accuracy, particularly when BIA is used to monitor subtle longitudinal changes in hydration or body composition. The validity of such calibration checks depends on the traceability of the reference components used.

Traceability is a core principle in metrology, referring to the ability to link a measurement result to a recognised reference standard through an unbroken chain of calibrations, each associated with a defined uncertainty. In clinical practice, this implies that BIA devices are calibrated using reference components traceable to national metrology institutes (NMIs) within the CIPM Mutual Recognition Arrangement (CIPM MRA), such as NIST in the USA [70] or NMIs within the EURAMET network in Europe [71].

This principle is well established in laboratory medicine, where test results (e.g., serum creatinine or glucose) are routinely compared with reference methods to ensure consistency across instruments and institutions [72]. Operationally, maintaining traceability requires clinics to select devices with documented calibration procedures, perform regular verification checks, and maintain accurate calibration records. Although some BIA devices include internal calibration routines, periodic external verification remains essential. Without traceability, systematic bias may go unnoticed, potentially compromising measurement quality, much as uncalibrated laboratory tests undermine diagnostic reliability.

In BIA, traceability applies to both the raw impedance data measurement and the physiological estimates derived from it, such as TBW or FFM. Electrical traceability is relatively straightforward: impedance measurements can be linked to the International System of Units (SI) through calibrated measurements of electric current (ampere) and time (second), from which voltage (volt) and resistance (Ω) are derived (Table 5). This SI-based traceability ensures that impedance measurements are physically meaningful and comparable across devices, studies, and clinical settings.

By contrast, the traceability of body composition estimates is more complex. Although FM is expressed in the SI unit kilogram, it cannot be measured directly. In practice, FM is therefore determined by comparison with established reference methods, such as DXA, hydrodensitometry, or isotope dilution, rather than through direct traceability to an SI standard. A BIA-derived estimate may be considered traceable to these methods if the device’s prediction equations have been calibrated against them, and if those reference methods are themselves traceable to fundamental physical measurements, such as mass, volume, or chemical concentration. For example, dilution methods for TBW are traceable to chemical analyses of isotope concentration, which are linked to standards of mass and volume.

Maintaining traceability and accuracy, therefore, requires that BIA devices be validated against appropriate reference methods for the specific populations in which they are intended for use. Validation claims are often based on comparisons with reference methods in limited study populations (e.g., DXA in N = 100 adults, *r* = 0.95, SEE = 2 kg for FFM). However, when devices are applied to different populations, such as children or patients with fluid imbalance (e.g., heart failure or dialysis), these validation results may no longer be applicable. In such cases, population-specific validation studies, adjusted prediction equations, or explicit awareness of potential bias are required. Where a consistent bias is known (e.g., systematic overestimation of FFM by 2 kg), correction factors may be applied or results interpreted accordingly, analogous to slope or offset corrections used in clinical chemistry.

Another aspect of traceability concerns the use of physical reference materials or phantoms. Unlike clinical biochemistry, where standard reference materials are widely available, BIA lacks universally accepted physical standards that mimic the electrical properties of the human body. Nevertheless, various phantoms have been developed for research and device testing. For example, one study [57] employed a jelly phantom with known electrical properties to evaluate device performance under controlled conditions. Although such phantoms are not yet commercially available, they may play an important role in future international standardisation by enabling cross-device and cross-site comparability [73].

If a device consistently measures a known standard within specification, its readings can be considered accurate (i.e., free of significant bias) within that range; otherwise, servicing or recalibration is required. Traceable calibration ensures that a given impedance value (e.g., 250 Ω) corresponds to the same physical quantity across devices, within a defined margin of uncertainty. This comparability is essential when pooling data across clinical sites or studies and underpins regulatory certification in both the EU and the USA. Manufacturers must demonstrate accuracy and repeatability by complying with recognised standards, including those issued by the International Electrotechnical Commission (IEC), and regulatory requirements from authorities such as the U.S. Food and Drug Administration (FDA). In the EU, conformity with the Medical Device Regulation (MDR, EU 2017/745) is typically achieved through adherence to harmonised standards [74]. From a regulatory perspective, metrological rigour also underpins compliance with ISO 13485 [75] quality management systems and the clinical performance evaluation requirements of the MDR, where documented calibration, uncertainty evaluation, and risk management form part of the technical documentation necessary to demonstrate analytical and clinical performance.

Metrological calibration is therefore not only a technical requirement but also a clinical and regulatory necessity.

## 5. Translating BIA Measurements into Clinical Insight and Action

### 5.1. Clinical Interpretation, Decision-Making, and Utility

Clinical decision-making inherently involves uncertainty [76,77], and quantitative tools such as BIA both quantify and introduce uncertainty that must be managed within a metrological framework.

Interpreting BIA results requires contextual understanding, including comparison with population-based reference values. PhA, derived directly from R and X_C_, reflects cellular membrane integrity and overall cellular health and has demonstrated prognostic value in multiple clinical studies [66,78]. In healthy adults, PhA values are typically observed approximately between 5° and 7° in women and 6° and 8° in men, depending on age and measurement conditions [66,79]. Values below the normal range are often associated with malnutrition, illness, or increased mortality risk, whereas higher values are frequently observed in athletes and physically active individuals and are not indicative of health deficiency, despite being outside typical reference ranges [51,80]. Equally important is the interpretation of intra-individual changes over time; for instance, whether FFM increases, remains stable, or decreases following a nutritional intervention [1,8].

Interpretation must also account for measurement uncertainty. Even if values differ between two time points, the observed change may fall within the device’s typical error margin and thus not reflect a true physiological shift. BIA uncertainty reflects a combination of technical, physiological, measurement-related, and modelling factors. Consequently, small changes over time should be interpreted cautiously, as they may fall within expected measurement variability despite appearing numerically precise. For example, TBW measured by whole-body BIA in healthy adults has been reported to have a standard error of approximately ±0.5 to ±1.0 L compared with isotope dilution methods [7]. A change from 30.0 to 30.3 L may therefore fall within expected measurement variability and remain below the MDC, making it unlikely to represent a true physiological change or reach the MCID. In contrast, a substantial reduction in FFM, for example, from 55 kg to 50 kg, would clearly exceed expected measurement variability and warrant clinical attention.

A common application of BIA is longitudinal tracking of body composition, such as monitoring muscle loss during illness or changes in hydration associated with heart or kidney failure. In this context, a key methodological question is what magnitude of change exceeds normal biological and measurement variability. This is captured by the concept of least significant change (LSC), which represents the minimal change required to be confident that an observed difference reflects a true physiological change rather than expected measurement variability [1,32].

To further operationalise the clinical interpretation of BIA within a metrological framework, Table 6 summarises selected clinical applications, commonly used BIA parameters, major sources of uncertainty, and recommended interpretation strategies.

The table is intended to support practical clinical interpretation by linking BIA-derived parameters to common clinical applications and their associated metrological limitations.

As described earlier, technical accuracy refers to the degree to which a measurement aligns with the reference value. Biological plausibility refers to the consistency of the measurement with other known aspects of the person’s physiology. BIA estimates may sometimes lack technical accuracy yet still convey useful biological information [81]. For example, raw impedance values can be used in BIVA, which plots R and X_C_ normalised for height. Even if absolute estimates of FFM are inaccurate, BIVA patterns can still provide qualitative indications of hydration status or cellularity [82].

In patients with fluid imbalance, conventional BIA equations may produce implausible results. For instance, acute fluid ingestion in healthy adults has been shown to skew BIA outputs, temporarily underestimating FFM and overestimating FM [83]. This illustrates clinical situations of fluid overload, in which BIA equations assume constant tissue hydration and thus misattribute excess fluid to tissue mass.

In contrast, BIVA more directly reveals vector displacement patterns consistent with states such as hyperhydration [12]. In such cases, it is more appropriate to trust the pattern rather than absolute numerical estimates, much like in ECG interpretation, where clinical emphasis is placed on recognising patterns such as rhythm, waveform morphology, and intervals rather than exact voltage amplitudes, which may vary with electrode placement or body composition.

This is where clinical judgment intersects with metrology: recognising when a measurement does not fit the overall clinical picture and reconsidering its interpretation, considering uncertainty, plausibility, and context. An example occurs in malnutrition or cancer cachexia, where BIA may underestimate muscle loss because increased hydration of the remaining lean tissue alters impedance-based estimates, thereby masking true reductions in lean tissue mass [8,9].

If a result appears biologically implausible, for example, when a patient shows clear muscle wasting but BIA reports normal muscle mass, the assumptions underlying the prediction are likely violated [1,7,9]. In such cases, the result should be interpreted with caution and, where possible, reviewed using alternative, physiologically informative parameters derived directly from raw impedance data, such as PhA [5,78]. In these applications, the clinical value of PhA lies less in conversion to body-composition units such as kilograms or litres and more in its relative value or change over time, which can convey meaningful biological information even when model-based estimates are unreliable [1,5,78].

Squara et al. [15] introduced the terms “alarm and titration”, referring to measurement-driven actions in critical care as part of a metrological framework for clinical decision making. An “alarm” signifies a rapid or unexpected change in a parameter that prompts clinical attention. In BIA, a sudden drop in low-frequency impedance, reflecting ECW, could signal fluid accumulation and potentially serve as an early warning of oedema.

“Titration” is the process of adjusting therapy incrementally based on measurements, such as modifying fluid or nutrition plans in response to daily or weekly BIA data. In both cases, defining what constitutes a meaningful change must consider biological and technical variability. For instance, if an alarm were set for a >2% drop in FFM, how often would normal variation trigger it? If it occurs too frequently, the threshold loses its utility.

By analysing historical data and test–retest error, more specific alarm triggers can be defined, for example, a sustained drop of more than 5% over 48 h might prompt a fluid retention alert. For titration, such as adjusting nutrition support, a clinician might consider a month-long increase in FFM as evidence of treatment efficacy. However, suppose the change is within the error margin or does not exceed the least significant change. In that case, they may choose to extend the observation period rather than adjust the plan prematurely. This conservatism helps prevent overreaction to measurement noise.

In practice, based on observed same-day (≤0.2 L) and between-day (≤0.5 L) variability in TBW, changes smaller than approximately ±0.5 L are likely within biological and measurement variability [47]. In contrast, an increase in TBW of ≈3 L over one week is well beyond expected measurement variability and may represent a clinically meaningful change warranting consideration of intervention if consistent with clinical findings, whereas a 0.5 L change would typically not prompt treatment adjustment.

By applying such quantitative reasoning, the risk of acting on false positives (i.e., apparent changes that are not real) can be reduced. Statistically, this represents the smallest change that exceeds normal measurement variability, estimated from repeated measurements and typically set at approximately 2 standard deviations (2 × SD). While the coefficient of variation (CV) is useful for comparing relative precision across methods or ranges, the standard deviation is the appropriate metric for assessing absolute change in each measurement.

Uncertainty also plays a role in the setting of diagnostic thresholds [84]. A single BIA measurement showing increased ECW may suggest fluid overload in a heart failure patient, but interpretation should account for baseline values and normal day-to-day variability. Serial measurements are often required to confirm meaningful trends and avoid overinterpreting normal physiological or technical variability [85]. This approach is well illustrated in lymphoedema assessment, where longitudinal changes in inter-arm ECW or resistance ratios are evaluated; for example, an increase exceeding 2 × SD in the inter-arm R_0_ ratio, defined as R_0 (affected)_/R_0 (contralateral)_, is commonly used to indicate lymphoedema [86].

Many clinical decisions rely on thresholds (cut-offs) to guide diagnosis, risk stratification, and treatment planning [84]. A low PhA has been associated with increased mortality risk in cancer patients, whereas reduced fat-free mass index (FFMI) is commonly used to identify sarcopenia [78,87].

When the uncertainty of a measurement is known, one can refine threshold-based decisions. This can be done by introducing grey zones with intervals between positive and negative thresholds, where test results are considered inconclusive [84], or by applying Bayesian thinking, which integrates prior clinical knowledge with new data to update the probability of a condition [88]. For illustrative purposes, suppose that a hypothetical clinical guideline based on expert consensus defines 17.0 kg/m^2^ as the sarcopenia threshold for FFMI in men. If a patient’s BIA-based FFMI is 16.5, the value is only 0.5 kg/m^2^ below the threshold, which is within typical measurement uncertainty. In contrast, a value of 14.0 kg/m^2^ would fall well below the threshold, even when measurement error is accounted for, and could therefore be considered abnormal.

To manage such uncertainty, a grey zone of ±2 SD around the threshold may be defined. In the example above, the threshold was 17.0 kg/m^2^ and SD = 0.5 kg/m^2^, giving a grey zone from 16.0 to 18.0 kg/m^2^. Many BIA-derived outputs are reported as precise numerical values (e.g., TBW = 34.5 L), which may create a false impression of absolute accuracy. Such estimates are derived from prediction models and remain subject to measurement uncertainty, biological variability, and model error. In some clinical applications, interpretation based on physiological status or risk categories may therefore be more robust than reliance on a single numerical estimate [89].Values in the grey zone may warrant additional confirmation, such as repeat BIA measurements under standardized conditions, longitudinal assessment, or use of a complementary method, before a diagnostic decision is made. This reduces the risk of misclassification. It is analogous to laboratory testing, in which results near decision thresholds or reference limits are often repeated or interpreted cautiously [90]. From a metrological perspective, this involves considering the measurement uncertainty interval around the clinical decision threshold. In laboratory medicine, this approach is increasingly used to support interpretation of results near decision limits, and some advanced laboratories explicitly communicate uncertainty in probabilistic terms (e.g., “16.5 ± 0.5, implying a defined probability that the true value lies below 17.0”) [27,91]. Clinical interpretation should never rely solely on BIA outputs. Instead, these measurements must be integrated with clinical findings, medical history, and supporting data.

Another way to reduce decision risk is to confirm important findings by repeating measurements or using an additional, complementary method. This approach minimises the impact of individual measurement errors, compensates for method-specific limitations, and enhances diagnostic confidence, particularly when a single method is inconclusive. For example, in dialysis care, BIA-derived estimates of target weight and overhydration are often interpreted alongside blood pressure trends and clinical assessment of oedema to support volume management decisions [92].

Each modality has distinct sensitivity and specificity for assessing volume status; therefore, bioimpedance measurements are typically interpreted alongside clinical findings and biomarkers in dialysis patients [92]. For example, BIA may quantify 2 L of excess fluid; if this aligns with clinical signs such as ankle oedema and elevated jugular venous pressure, the evidence becomes consistent and clinically actionable, supporting the decision to remove fluid [93].

As a further example, BIA may indicate substantial excess fluid in patients who appear clinically euvolemic; in such cases, results should be interpreted cautiously and in conjunction with other clinical information rather than acted upon in isolation [92]. Here, uncertainty extends to the system level: concordant findings across independent methods increase confidence, whereas discordant results highlight the need for further evaluation [15]. This approach is consistent with metrological principles [27], including cross-validation using independent methods.

Ultimately, the usefulness of BIA lies in whether it improves clinical decisions or outcomes [94]. Highly accurate reference methods may be impractical for routine clinical use because they are costly, time-consuming, or unsuitable for frequent repetition. In contrast, a precise but moderately accurate method such as BIA, which is accessible, non-invasive, and repeatable at the bedside, can be highly valuable for monitoring change over time.

For instance, BIA-derived measures of muscle mass are commonly used to identify sarcopenia in older individuals [87]. Furthermore, this is particularly true in settings such as intensive care or dialysis, where frequent and dynamic assessments are crucial. Daily DXA scans are impractical in the intensive care unit (ICU), but BIA enables bedside monitoring of fluid status. Outcome-oriented studies suggest that BIA-guided interventions can be beneficial; for example, adjusting fluid removal in dialysis patients based on BIA-derived volume assessment, or using phase angle to inform nutritional support in oncology [92,95].

These examples underscore the importance of evaluating BIA not only against reference methods, assessing accuracy and precision, but also against its impact on patient outcomes, including whether it guides treatment, reduces complications, or improves quality of care, for instance in randomized trials comparing BIA-guided management with standard care. Recommended study designs for such evaluations are summarised in Table 7, which also illustrates how clinical utility can be systematically assessed.

From a metrological perspective, this reflects the principle of *appropriateness for its intended purpose*: ensuring that measurements are not only accurate but also practically relevant to clinical decision-making. BIA may not meet the accuracy needs of all applications (e.g., pharmacokinetics). Still, for tasks such as tracking hydration status, indicating sarcopenia, or screening for obesity, its measurement uncertainty may be well within acceptable bounds. Even when advanced BIA modelling is unreliable due to acute illness, as in critically ill patients, raw impedance metrics such as R and PhA often retain clinical relevance [96].

By combining good measurement practice with an understanding of physiological context and statistical variation, BIA becomes an effective tool for better patient care.

### 5.2. Method-Specific Limitations and Validity

Validation of BIA devices or prediction equations is commonly performed by comparing their body-composition outputs with those of a so-called gold-standard technique. For body-composition analysis, methods such as underwater weighing (densitometry), DXA, air-displacement plethysmography (ADP; BodPod), and isotope dilution (e.g., deuterated water or sodium bromide) are commonly used as reference techniques [2]. However, from a metrological perspective, the concept of a “gold standard” is not a formal metrological term and can be misleading [15]. The International Bureau of Weights and Measures (BIPM) defines reference measurement procedures and reference materials that provide results accepted as fit for their intended purpose [17]. Accordingly, the term “gold standard” is used informally in clinical practice to denote the best available method rather than an absolute reference [15].

However, reference methods also have limitations: they introduce measurement uncertainty and may not assess the same underlying construct as the method being evaluated, such as BIA [97]. When comparing BIA with reference methods, several issues should be considered [1,15,28,97]:

- BIA and the reference method may be assessing different things.
- Reference methods themselves are subject to measurement uncertainty.
- Agreement is sometimes assessed using simplistic metrics, such as correlation coefficients, which reflect linear association rather than agreement; more informative approaches include assessment of bias and LoA using Bland–Altman analysis.
- Even if BIA shows lower absolute accuracy, it may still be clinically useful if its errors do not compromise the intended application, such as trend monitoring or broad classification.

Table 8 presents commonly used terms in body composition assessment and BIA. Terms used to describe established comparison procedures are often applied interchangeably, despite important conceptual differences. Distinguishing between them enhances clarity in the evaluation of measurement validity.

BIA is commonly interpreted at the output level as a two-compartment (2C) model separating FM and FFM. This separation is based primarily on measurements of body water, since the conduction of electrical current depends largely on TBW. At the measurement level, however, BIA is frequency-dependent: low frequencies predominantly reflect ECW, whereas higher frequencies reflect TBW; accordingly, MF-BIA and BIS explicitly model ECW and ICW before deriving FFM estimates [3]. Because water is primarily contained within FFM, BIA assumes that higher electrical conductivity reflects a greater amount of lean tissue [1].

In normally hydrated individuals, BIA-derived estimates of FFM generally correspond to non-fat soft tissue mass (e.g., muscle and organs) but exclude bone mineral, in contrast to DXA, where body weight is partitioned into FM, lean soft tissue, and bone mineral content [100]. However, this assumption can lead to overestimation of fat-free mass in individuals with fluid retention, such as those with oedema or inflammation, because BIA cannot distinguish between water contained within muscle tissue and excess ECW, which may therefore be misclassified as part of FFM, a limitation well recognised in both methodological analyses and clinical studies of overhydrated patient populations [1,39,92]. Similarly, DXA, which derives soft-tissue composition from X-ray attenuation, may interpret excess fluid as an increase in lean soft-tissue mass, since fluid contributes to the total soft-tissue signal despite not being structurally part of muscle or organ tissue [100].

Understanding these distinctions is essential:

- BIA-derived FFM reflects tissues that contain water and conduct electricity, primarily in muscle and internal organs.
- DXA-derived lean soft tissue mass represents all non-fat, non-bone soft tissues and is therefore sensitive to abnormal fluid accumulation, although it remains an indirect estimate compared with anatomically defined methods such as MRI or CT.
- Excess fluid, while not functional tissue, is difficult to distinguish from true lean tissue in both methods.

Both methods may indicate an increase in lean mass, even when this primarily reflects fluid accumulation rather than a true gain in functional tissue. In body-composition terminology, FFM comprises all non-fat components, including TBW (both ICW and ECW), but excludes bone mineral. Consequently, excess fluid can legitimately increase FFM, even if it does not represent an increase in muscle mass or cellular tissue [3,7,50].

This illustrates that what may appear to be a measurement error may instead reflect differences in the quantity being quantified. A related example concerns differences in how bone mineral and lean tissue are quantified across body-composition methods. BIA does not directly assess bone mass, as bone contains little water and contributes minimally to electrical conductivity; bone is therefore implicitly incorporated into FFM in BIA-based models [3,38]. DXA, by contrast, quantifies bone mineral separately and estimates lean soft tissue mass based on attenuation differences between bone and non-bone tissues, with FFM subsequently derived as the sum of lean soft tissue and bone mineral content [60,98].

In individuals with unusually high bone mass, BIA may therefore underestimate FFM relative to DXA, not because the measurement is incorrect, but because the two methods define and quantify FFM differently [1,3,98,100]. The key implication is that comparisons between methods must recognise these conceptual differences, rather than treating all techniques as if they measure the same type of “lean mass”.

Short-term repeatability of total body fat percentage measured by DXA is commonly reported to be on the order of 1–2% (coefficient of variation) under standardized conditions [101], although precision varies by anatomical region and device platform, and systematic differences may arise from scanner technology, model, and segmentation algorithms [102].

Hydrodensitometry, and its modern counterpart air-displacement plethysmography (ADP, e.g., Bod Pod), assume constant tissue densities, typically 0.9 g/cm^3^ for FM and 1.1 g/cm^3^ for FFM, and rely on precise body-volume determination (e.g., complete exhalation in hydrodensitometry) [98]. However, these assumed densities are not constant across individuals and may vary due to differences in bone mineral content, protein composition, and hydration status, thereby introducing systematic error into densitometric estimates.

Isotope dilution techniques, using deuterated water (D_2_O) to measure TBW and sodium bromide (NaBr) to measure ECW, assume uniform tracer distribution and complete equilibration, as described in standard methodological references [98]. However, they are also subject to analytical and procedural measurement errors during sample collection and analysis [98].

When BIA is reported to differ from DXA by, for example, ≈5% body fat, this difference should not be attributed to BIA alone. Part of the discrepancy reflects the uncertainty and methodological assumptions inherent in the reference method, including DXA [61]. Although DXA is often treated as a reference method, it has known precision limits and systematic biases related to scanner technology and tissue segmentation, which contribute to uncertainty in body-fat estimates [103].

Because all body-composition methods involve assumptions and uncertainty, differences between BIA and a reference method do not necessarily reflect error in BIA alone; part of the discrepancy may arise from uncertainty in the reference method itself [1,27,29,97].

This highlights the need for a more rigorous approach to method comparison, one that estimates total error by combining the uncertainties of both methods. For instance, if DXA has a precision error of ±2% and BIA ±3%, then assuming independent random errors, the combined uncertainty can be approximated using root-sum-of-squares propagation as $\pm \sqrt{2^{2}+3^{2}}\approx \pm 3.6\%$. Table 9 summarises the major contributors to uncertainty in BIA-derived body composition assessment, including both random and systematic sources of variability and bias. The table focuses on uncertainty sources most consistently identified in the literature as clinically and methodologically important, although additional physiological, procedural, environmental, and device-related factors may also influence BIA measurements and their interpretation, particularly when measurement conditions are insufficiently standardised.

This result is obtained by combining independent errors using the root-sum-of-squares formula above, which accounts for the accumulation of random measurement uncertainties [27].

BIA often shows strong correlations with reference methods, such as DXA or deuterium dilution, when measuring FFM or TBW [1]. Many early validation studies used correlation coefficients, which are insensitive to bias and typical error magnitudes, as indices of method comparability [15]. Current recommendations suggest using LoA analysis (Bland–Altman plots) to assess bias, along with 95% LoA [28], and adding metrics such as the SEE and total error. Validation studies often report agreement in terms such as % body fat by BIA = DXA ±5% (95% LoA). While this level of error may be unacceptable for applications requiring high individual precision (e.g., research-grade body composition assessment), it may be clinically acceptable for obesity screening [1,7].

At the group level, mean differences between BIA and reference methods are typically small (≈1–2%), whereas at the individual level, discrepancies are substantially larger, commonly in the range of ±5–10%, as demonstrated using Bland–Altman analyses [1,28]. This indicates that while BIA-based prediction equations perform well at the population level, individual estimates may differ noticeably due to combined methodological and inter-individual physiological variability [40,51].

Systematic biases in BIA and DXA may act in opposite directions, such that overestimation by one method coincides with underestimation by the other, producing apparent agreement despite underlying error in both. In a controlled study using an MF-BIA device, an initial underestimation of body fat percentage of approximately 4% relative to DXA was observed. Applying a post hoc +3% correction reduced the bias to −1.0 ± 2.8%, improving agreement (r = 0.932; CCC = 0.920) without loss of precision [47].

For example, one study reported that BIA underestimated body fat percentage by approximately 4 percentage points relative to DXA, with a LoA of approximately ±5.6 percentage points [47]. Although such differences may be relevant for precise quantification, they may not compromise broader clinical applications, such as classifying individuals as obese using a 25% body fat threshold or monitoring changes over time, provided the bias is consistent. In this context, consistent systematic bias can be acknowledged or adjusted for; in the same study, applying a simple +3% correction substantially improved agreement [47].

### 5.3. Communicating Measurement Uncertainty in Clinical BIA Practice

In clinical practice, communication of measurement uncertainty is challenging, particularly when results are presented as a single numerical value (e.g., 45.1 L TBW), which may convey a precision that is unwarranted [27]. Greater transparency can be achieved by expressing results in a manner that reflects their inherent uncertainty. For example, instead of reporting a patient’s body fat as 30.2%, the result could be communicated as approximately 30%, with an uncertainty of ±3 percentage points, corresponding to an approximate expanded uncertainty (≈95% coverage) rather than a single standard deviation, in line with established metrological principles [27].

Even when uncertainty is conveyed qualitatively, such as noting that results should be interpreted within a few percent margin of error, this approach can help avoid overprecision in reporting and support appropriate clinical interpretation [91]. Some BIA devices report internal indicators intended to reflect model stability or estimation confidence. As these indicators are derived from proprietary, device-specific algorithms and are not standardised, they should be interpreted cautiously [1]. When available, this information could be included in the clinical documentation to support interpretation. The goal is to ensure that the end user, such as a clinician or dietitian, has the necessary context to interpret the results appropriately.

Small absolute differences are not necessarily clinically meaningful if they fall within ranges associated with similar physiological states. Instead, clinical relevance depends on the relative magnitude and context of the change. For example, a 1 kg difference in FM represents only a 4% change at 25 kg but a 10% change at 10 kg, leading to different clinical interpretations. Likewise, modest differences in PhA within a low range may not affect decision-making, whereas larger shifts across established reference ranges reflect distinct physiological states (e.g., malnutrition versus athletic conditioning), as shown by population reference values and associations with physical activity [79,80].

From a broader perspective, integrating metrological principles into medical practice contributes to patient safety. A substantial proportion of patient harm in diagnostic and monitoring settings has been attributed to faulty measurements, inadequate consideration of uncertainty, or misinterpretation of quantitative results [111,112]. A 2024 study in JAMA Internal Medicine [113] found diagnostic errors in 25% of hospitalised patients who died or were transferred to the ICU, most of which caused harm.

By systematically reducing measurement errors through calibration, maintenance, proper training, standardisation, and acknowledging the remaining uncertainty, it is possible to make decisions with appropriate caution, thereby reducing the risk of misinformation or inappropriate interventions. In the context of BIA, misestimating a patient’s FFM and thus under- or over-prescribing specific interventions could lead to inappropriate nutritional support, e.g., overfeeding a patient whose lean mass is overestimated, potentially worsening metabolic stress. Similarly, in heart failure management, underestimating fluid overload due to a misread BIA value might fail to initiate timely diuretic therapy. These issues may be avoided if one follows the principles discussed.

Finally, risk management using metrology also involves what to do when a measurement does not make sense. A single abnormal BIA reading, such as a sudden increase in TBW that seems physiologically impossible, should prompt troubleshooting: check the electrodes, repeat the measurement, and consider whether a change has occurred, e.g., did the patient consume a large amount of water? This is analogous to the stringent quality control adopted in laboratory medicine. If a control test fails, one cannot trust that day’s results until the failed test is resolved. By building such a mindset, clinicians can use BIA as a reliable tool, but one whose output is always subject to verification and clinical context.

### 5.4. Best Practices for BIA Measurement and Implementation

By integrating the metrological concepts and thoughts presented in this paper, it is possible to develop practical guidelines for implementing BIA in both clinical and research settings. Such guidelines should serve as a checklist to ensure high-quality measurements and interpretations that align with the best metrological practices and clinical utility. Below is a structured and coherent set of guidance for best practices for BIA measurements.

- *Establish a standardised measurement protocol*

Develop a written protocol for BIA measurements and ensure that all personnel are trained in its use. The protocol should cover patient preparation (including fasting and activity restrictions), electrode placement, body positioning, and device operation. Having a standard operating procedure (SOP) reduces variability and improves reproducibility in adults [7] and children [14,46].

- *Standardise patient preparation*

Instruct patients on pre-test requirements. A typical set of pre-measurement instructions includes: “Avoid eating or drinking for at least 4 h before the test; abstain from alcohol for 24 h before the test; refrain from vigorous exercise for 8 to 12 h before the test; and empty the bladder approximately 30 min before the measurement”. Measure under similar conditions each time (e.g., at the same time of day). Document any deviations (e.g., if a patient had to drink water due to thirst) so results can be interpreted accordingly.

- *Standardise electrode placement and posture*

When using lead-type BIA devices, mark the exact electrode sites (especially in research studies) or use anatomical landmarks to ensure accurate placement. Ensure good contact; if using adhesive electrodes, clean and dry the skin. If using devices with built-in stainless-steel electrodes, ensure the skin is clean, dry, and free of excessive callus where possible. When thickened skin is unavoidable (e.g., on the hands or feet), consider conductive gel, alternative electrode placement (if supported), or documentation of reduced signal quality [114].

Maintain the required posture, i.e., supine for devices designed for this position, or standing as appropriate. If supine, allow ≈5–10 min resting to stabilise fluids before measurement. If standing, ensure the person is centred and still, arms slightly away from the sides.

- *Calibrate and maintain the device*

Follow the manufacturer’s calibration routines. If the device has a self-check or calibration mode, use it routinely, such as daily in research use or weekly in clinical use. Periodically test the device using the manufacturer-provided calibration resistor. Keep the device clean and inspect electrodes and cables for any damage or corrosion. Replace electrode pads for each patient (if disposable) or clean reusable electrodes regularly. Keep firmware updated if the manufacturer issues improvements, especially if they correct algorithmic biases. Calibration and maintenance activities should be documented in accordance with standard procedures for laboratory instruments.

- *Apply appropriate population-specific equations*

Ensure that the BIA device or software uses equations suitable for the individual’s characteristics. Use paediatric-specific BIA equations for children; adult formulas can significantly misestimate body composition in children [115]. Likewise, apply obesity-specific equations in severely overweight populations [116]. Wherever possible, select device presets or algorithms appropriate to the patient’s characteristics, such as child or obese modes, to enhance estimation accuracy. If a device does not offer a suitable option, consider applying published equations manually or using an alternative device. Misapplication of equations is a significant cause of BIA inaccuracy [81], a problem further compounded by the limited transparency of proprietary algorithms in many commercial devices [117], which may hinder traceability and the appropriate interpretation of results.

- *Recognise device limitations and validate when needed*

If BIA is used in a new context, such as with a different ethnic group or a disease state, try to validate a subset of measurements against a reference method. Even a small study comparing BIA to DXA or dilution in the clinical setting can reveal biases. For example, if the patient population consistently shows a +2 kg bias in FFM, it is possible to adjust for that. Use validation studies of the specific device (and algorithm) in populations like those in the study. In research reporting, specify the device model and algorithm/software version and report repeatability metrics based on repeated measurements (e.g., duplicate or triplicate measures), alongside any available accuracy data [47].

- *Account for measurement uncertainty in interpretation*

When reviewing results, consider the known error margins. Do not over-interpret small changes. For individual monitoring, define thresholds for meaningful change based on the device’s known repeatability, biological variability, and clinical context rather than fixed universal cut-offs. For single measurements around a clinical cutoff value, use clinical judgment and consider repeating the measurement if the value is uncertain. Performing three consecutive measurements and confirming that they fall within a narrow range can help verify reliability; discrepancies may indicate a technical error or physiological instability.

- *Interpret raw impedance data in context*

Do not automatically trust the device’s output (such as % body fat) without considering whether it’s accurate or suitable for that patient. Monitor the raw impedance (Z, R, X_C_) values, if possible. These parameters indicate whether a change is due to hydration (a significant drop in R) or to something else.

For example, in a patient, a marked reduction in X_C_ (and thus PhA) may reflect loss of cell mass or altered fluid distribution, even in the absence of weight change, and may therefore serve as a potential early indicator of clinical deterioration [12]. Thus, raw impedance data, combined with clinical observation, can provide deeper insight than black-box outputs, such as % body fat or muscle mass, and support more informed decision-making.

- *Integrate BIA into a holistic clinical assessment*

Avoid making significant decisions based solely on BIA. It should augment clinical judgment, not override it. If BIA indicates a surprising result, e.g., very low muscle mass in someone who appears muscular, double-check and verify the measurement.

When using BIA to guide therapy (such as fluid removal), use it in conjunction with vital signs and clinical signs. Recognise that BIA provides estimates that should be triangulated with other evidence.

- *Ensure transparent documentation and reporting*

In research publications, important measurement details should be reported, including the BIA device used, software version, measurement frequencies, electrode placement, calibration status, prediction equation, and measurement conditions such as fasting status, posture, and recent physical activity. Reporting measures of measurement consistency and uncertainty, when available, may further improve transparency and comparability between studies and clinical settings. In clinical records, note any factors that might have affected the reading, for example, “measured after dialysis session” or “patient had ascites present”. Transparency helps others interpret the data correctly and ensures continuity. This allows subsequent clinicians to assess whether subsequent measurements were obtained under similar conditions.

By following these principles and recommendations, common sources of error in BIA may be reduced, thereby improving the reliability, reproducibility, and clinical usefulness of measurements and their interpretation. Key recommendations are summarised in Table 10.

## 6. Conclusions

BIA exemplifies how a medical measurement technique can offer valuable insights into body composition while also presenting challenges that require careful control and interpretation. By examining BIA through the lens of metrology, this paper has highlighted that obtaining a numerical value is only one part of the measurement process.

Ensuring that values are meaningful, accurate, and actionable requires attention to the complete measurement-to-decision chain. This includes clearly defining the measurand (the specific quantity and conditions being measured), ensuring reliable measurement through calibration and standardised protocols, and understanding and quantifying uncertainty. Finally, the result must be thoughtfully integrated into the clinical or research context. When applied to BIA, metrology in medicine offers several important insights. First, core measurement concepts, such as accuracy, precision, bias, repeatability, and uncertainty, are not abstract; they directly influence how BIA is performed and how its outputs are interpreted. Accuracy may be compromised if devices are not calibrated or if inappropriate prediction models are applied, while standardising protocols and controlling conditions can enhance precision. Second, every BIA measurement is influenced by multiple factors, some of which are controllable, such as posture or electrode placement, and others that must be accounted for, such as biological variability. By systematically identifying and addressing these sources of uncertainty, the risk of drawing misleading conclusions can be reduced. Third, the conventional notion of validation against a so-called “gold standard”, often operationalised in practice as comparison with a reference method, warrants a more nuanced view.

While validation is necessary, reference methods themselves have limitations, and clinical usefulness should remain the ultimate benchmark. In this context, a method’s clinical applicability becomes a central consideration.

BIA may therefore be suitable for specific clinical or research purposes, provided it is used appropriately and within its established limitations. Accordingly, the importance of traceability and standardisation must also be emphasised, as these are essential for building confidence in BIA-derived measurements.

A culture of quality, treating BIA devices with the same rigour as other medical and laboratory instruments used in clinical settings, can produce data that clinicians can interpret and act upon with greater confidence. Conversely, neglecting such principles risks reducing BIA to a black-box tool generating numbers of uncertain value.

Without appropriate calibration, standardisation, traceability, uncertainty awareness, and clinical interpretation, BIA risks functioning as a “black box” technology that produces numerical outputs with unclear physiological and clinical meaning.

As the field of body composition assessment advances, broader efforts to standardise BIA internationally will be required. These may include improved reference phantoms, cross-calibration protocols, and greater transparency from manufacturers regarding their algorithms and error margins. For clinicians and researchers, the practical guidelines and best practices outlined here can support more effective and appropriate integration of BIA into clinical workflows.

When used with methodological care and contextual understanding, BIA can make a meaningful contribution to patient care, whether supporting fluid management in different diseases, evaluating nutritional status in oncology, or monitoring fitness trends in preventive health. Its utility depends not just on technical performance, but on interpretation grounded in measurement principles and clinical reasoning.

Ultimately, the discipline of metrology offers both the mindset and methodological foundation to strengthen the role of BIA as a clinical measurement tool. Although limitations remain, transitioning from measurement to decision-making with scientific reflection and transparency enables more robust conclusions, both in patient care and in research. By embracing this approach, the reliability of BIA-derived data can be enhanced, thereby ensuring that decisions informed by those data are well-founded and clinically meaningful.

## Figures and Tables

**Table 1:** Overview of BIA techniques by technical and clinical characteristics.

Characteristics	SF-BIA	MF-BIA	BIS
Frequency and range	Single frequency (typically 50 kHz).	Multiple fixed frequencies (e.g., 3–6 discrete frequencies such as 5, 50, and 200 kHz).	Continuous spectrum (e.g., 2–1000 kHz).
Modelling approach	No spectral modelling (empirical regression-based estimation only).	Empirical estimation of fluid compartments.	Cole model + Hanai mixture theory.
Measured parameters	Z, R, X_C_ (if the device is phase-sensitive) at a single frequency.	Discrete R, X_C_, Z values.	Full impedance spectrum with curve fitting.
Clinical use	Estimates general body composition (e.g., TBW, FFM)	Assess general body composition plus ECW/ICW, hydration status.	In addition to overall body composition, it provides information on body water compartments (ECW/ICW) and cellular properties associated with inflammatory or disease-related changes.
Primary application	Screening and basic body composition estimation in healthy populations or primary care.	General body composition with hydration and fluid status assessment (e.g., oedema, dehydration, dialysis).	General body composition with detailed fluid compartment analysis (ECW/ICW), particularly where precise fluid monitoring is required (e.g., oncology, geriatrics, critical care).
Key limitations	Strong dependence on fixed FFM hydration and population-specific equations.	Still relies on model assumptions; limited resolution of intracellular changes.	Sensitive to measurement noise due to multi-frequency curve fitting; requires careful standardisation.

Overview of BIA techniques, comparing SF-BIA, MF-BIA, and BIS with respect to technical principles, modelling approaches, measured parameters, clinical applications, and key limitations. The table highlights the increasing complexity and physiological specificity of the techniques, along with differences in clinical utility and methodological requirements.

**Table 2:** Overview of common BIA parameters by measurement type and technique.

Parameter	Definition	Data Type	Method of Derivation	Applies to
Z	Electrical impedance: a frequency-dependent complex quantity describing opposition to alternating current.	Measured	Measured directly by the device as complex impedance (Z = R + *j*X_C_); devices may additionally report the impedance magnitude $\left|Z\right|=\sqrt{R^{2}+{X_{C}}^{2}}$.	SF-BIA, MF-BIA, BIS
R	Resistance: opposition to flow through body fluids (mainly ECW).	Measured	Measured directly by the device.	SF-BIA, MF-BIA, BIS
X_C_	Capacitive reactance: reflects the capacitive behaviour of cell membranes.	Measured	Measured directly by the device.	SF-BIA, MF-BIA, BIS
PhA	Phase angle: an indicator of membrane health and cellular integrity.	Generally derived	Derived by the device; $PhA=arctan\left(\frac{X_{C}}{R}\right)$ in degrees.	SF-BIA, MF-BIA, BIS
R_0_ (or R_E_)	Resistance at 0 Hz: derived from Cole modelling; used to estimate ECW.	Modelled	Determined from the Cole model.	BIS
R_I_	Intracellular resistance: derived from Cole modelling; used to estimate ICW.	Modelled	Estimated from the Cole model (calculated from R_E_ and R_∞_).	BIS
R_∞_ (or R_INF_)	R_∞_, derived from Cole modelling, represents current flow through extracellular and intracellular pathways and is used to estimate TBW.	Modelled	Determined from the Cole model.	BIS
C_m_	Cell membrane capacitance: reflects the ability of the membranes to uphold a charge difference and can be used to indicate cell membrane integrity.	Modelled	Estimated from the Cole model.	BIS
TBW	Total body water: sum of ICW and ECW.	Predicted	Estimated using regression (typically SF-BIA and MF-BIA) or Cole modelling and mixture theory (BIS).	SF-BIA, MF-BIA, BIS
ECW	Extracellular water: water outside cells.	Predicted	Estimated using regression (typically SF-BIA and MF-BIA) or Cole modelling and mixture theory (BIS).	MF-BIA, BIS
ICW	Intracellular water: water inside cells.	Predicted	Estimated using regression (typically SF-BIA and MF-BIA) or Cole modelling and mixture theory (BIS).	MF-BIA, BIS
FFM	Fat-free mass: non-fat components of the body.	Predicted	Estimated using regression or from TBW using an assumed hydration fraction for FFM.	SF-BIA, MF-BIA, BIS
FM	Fat mass: total fat content.	Predicted	Estimated using body-weight-FFM for the whole body or proprietary models.	SF-BIA, MF-BIA, BIS
BCM	Body cell mass: metabolically active tissue mass.	Predicted	Estimated from ICW or multi-compartment modelling; conceptually linked to intracellular potassium (e.g., via whole-body ^40^K counting in reference methods).	BIS

This table classifies standard BIA parameters by type (measured, derived, modelled, or predicted) and by BIA modality. Impedance (Z) is measured as a complex quantity, whereas parameters such as membrane capacitance (C_m_) are specific to BIS. Availability of R, X_C_, and phase angle (PhA) depends on phase-sensitive measurement. Some SF-BIA devices report only |Z|, while some MF-BIA devices report R, X_C_, and PhA at a single frequency (typically 50 kHz) despite multi-frequency acquisition.

**Table 3:** Overview of metrological and clinical-performance parameters in BIA evaluation.

Category	Parameter	Definition
Instrument performance	Resolution	The smallest change in input that produces a detectable change in output.
	Zero (offset) drift	Systematic change in baseline output over time without a change in input.
	Sensitivity (instrument)	Ratio of output change to input change.
	Measurement range	Interval between minimum and maximum values over which performance is specified.
	Linearity	The degree to which output is proportional to input across the measurement range.
	Frequency response	Variation in measurement performance across different input frequencies.
	Fidelity	The degree to which the device reproduces the amplitude, shape, and timing of the input signal without distortion.
Accuracy and validity	Accuracy (qualitative concept)	Closeness of agreement between a measured value and a reference value.
	Measurement error	The difference between a measured value and a reference value.
	Relative measurement error (%)	Measurement error expressed as a percentage of the reference value.
	Validity	The extent to which a measurement method is appropriate and meaningful for its intended purpose, population, and context of use.
	Limits of agreement (LoA)	Range within which approximately 95% of differences between two measurement methods are expected to lie (Bland–Altman analysis), reflecting agreement between methods.
Precision, variability and uncertainty	Standard deviation (SD)	A measure of the dispersion of repeated measurements around their mean.
	Coefficient of variation (CV, %)	Standard deviation expressed relative to the mean value.
	Repeatability	Precision under identical conditions (same operator, device, and short time interval).
	Reproducibility	Precision under changed conditions (e.g., different operators, instruments, or times).
	Technical error of measurement (TEM)	Within-method variability due to technical factors during repeated measurements.
	Measurement uncertainty	Parameter characterising the dispersion of values that could reasonably be attributed to the measurand.
	Least significant change (LSC)	Minimum change required between two measurements to exceed expected measurement variability (commonly ≈ 2 × SD or 1.96 × √2 × SEM).
	Minimal detectable change (MDC)	The smallest change that exceeds measurement error with a specified confidence level.
Agreement and Association	Pearson’s correlation coefficient (r)	A measure of linear association between two variables.
	Intraclass correlation coefficient (ICC)	A measure of reliability assessing agreement within grouped or repeated measurements.
	Cohen’s kappa (κ)	Measure of categorical agreement beyond chance.
Types of error	Standard error of estimate (SEE)	Standard deviation of residuals from a regression model; reflects prediction error.
	Bias (systematic error)	Mean difference between measured values and reference values.
	Random error	Unpredictable variation affecting repeated measurements.
Diagnostic Performance	Sensitivity	Probability that a test correctly identifies true positives.
	Specificity	Probability that a test correctly identifies true negatives.
	Positive predictive value (PPV)	The probability that a positive test result reflects the true presence of a condition.
	Negative predictive value (NPV)	The probability that a negative test result reflects the true absence of a condition.
Clinical relevance and decision-making	Minimal clinically important difference (MCID)	The smallest change in a measurement perceived as meaningful by patients or clinicians.
	Traceability	Property of a measurement result whereby it can be related to a reference standard (typically SI units) through an unbroken chain of calibrations, each with stated uncertainty.
	Number needed to treat (NNT)	The number of patients who must receive an intervention to prevent one additional adverse outcome.

This table summarises commonly used parameters for evaluating BIA devices and organises them by their relevance to measurement quality, clinical interpretation, and technical performance.

**Table 4:** Typical effects of measurement conditions on BIA parameters.

Parameter	Supine	Standing	De- Hydration	Over- Hydration	Post- Exercise	Post-Prandial
R	↓	↑	↑	↓	↓ or ↔	↓ or ↔
X_C_	↑ or ↔	↓ or ↔	↑ or ↔	↓ or ↔	↓	↓
PhA	↑	↓	↑	↓	↓	↓
Z	↓	↑	↑	↓	↓ or ↔	↓ or ↔
ECW/TBW ratio	↓	↑	↓ or ↔	↑	↑	↑

The expected directional changes shown in the table are based on established physiological principles and on the synthesis of the BIA literature [3,49]. The table presents a conceptual summary of physiological effects rather than a quantitative synthesis of evidence. Arrows indicate relative increases (↑), decreases (↓), or no consistent change (↔) relative to a standardised resting baseline (e.g., supine, well-hydrated, fasted). Parameter definitions are provided elsewhere. Trends represent generalised responses and may vary between individuals.

**Table 5:** The seven SI base units.

Physical Quantity	SI Unit	Abbreviation
Length	metre	m
Mass	kilogram	kg
Time	second	s
Electric current	ampere	A
Temperature	kelvin	K
Amount of substance	mole	mol
Luminous intensity	candela	cd

These base units define the International System of Units (SI). In BIA, electrical impedance is derived from measurements of electric current (ampere) and time (second), from which voltage and resistance are obtained, enabling calibration traceable to SI standards. Other SI units are indirectly relevant through reference methods and measurement conditions (e.g., mass in body composition assessment, amount of substance in isotope dilution, and temperature effects on conductivity), supporting comparability and reliable interpretation of BIA measurements.

**Table 6:** Clinical interpretation and uncertainty framework for selected BIA applications.

Clinical Setting	Primary BIA Outputs	Clinical Objective	Major Uncertainty Source	Recommended Interpretation Strategy
Heart failure	ECW, ECW/TBW, IR, BIVA	Fluid monitoring	Truncal insensitivity	Serial trend monitoring
Dialysis	BIS-derived ECW, ICW, R_0_, R_I_, R_∞_, BIVA	Dry-weight estimation	Hydration variability	Standardized timing
Oncology	PhA, X_C_, IR, potentially C_m_, BIVA, FFM	Nutritional and prognostic assessment	Inflammation and fluid shifts	Combining with the clinical context
ICU/Critical care	IR, X_C_, PhA, BIVA, potentially C_m_	Monitoring fluid redistribution	Extreme physiology	Interpret together with clinical and haemodynamic assessment

This table illustrates how sources of uncertainty may influence the interpretation of selected BIA parameters across different clinical settings.

**Table 7:** Recommended study designs for validating clinical endpoints related to BIA.

Clinical Endpoint	Purpose of Measurement	Recommended Study Designs
Morbidity (e.g., complications, infection)	Assess risk prediction or association with poor outcomes	Prognostic cohort study, observational cross-sectional study, randomized controlled trial (RCT evaluating BIA-guided care)
Mortality (all-cause or disease-specific)	Determine the predictive value of BIA-derived variables	Prospective cohort study, survival analysis, registry-based study
Treatment response (e.g., nutrition, drugs)	Evaluate the impact of BIA-guided monitoring on intervention outcomes	RCT evaluating BIA-guided treatment, pre–post intervention study, controlled trial
Hydration/fluid balance (e.g., overhydration)	Assess BIA’s clinical accuracy in fluid status assessment	Diagnostic accuracy study, comparison with dilution or clinical exam; RCT evaluating BIA-guided fluid management
Healthcare utilisation (e.g., readmission)	Assess BIA’s value for predicting or reducing system burden	Pragmatic trial, implementation study, retrospective cohort
Nutritional or functional status	Track or guide interventions targeting muscle mass or nutrition	RCTs evaluating BIA-guided nutritional interventions, longitudinal observational study
Procedure-related complications	Predict clinical complications (e.g., pressure ulcers)	Prognostic cohort study, RCT, quality improvement, or implementation study

This table outlines appropriate study designs for evaluating whether BIA-derived metrics can predict, monitor, or influence critical clinical outcomes. These designs support the clinical translation and evidence base for BIA beyond simple method comparison.

**Table 8:** Terminology in body composition method comparison and validation.

Term	Definition	Example	Relation to BIA	Comments
Gold standard	The best available method is commonly assumed to be most accurate in estimating body composition, although it is not necessarily a true reference.	DXA, 4- or 5-compartment model *	Used as a comparator to assess BIA performance.	Not a formal metrological term, although widely used in clinical literature. DXA has its own measurement uncertainty, and using it as a reference may attribute error to BIA.
Criterion method	A trusted method used to evaluate the validity of another technique.	DXA, isotope dilution	Used to assess the criterion validity of BIA estimates.	Often overlaps with “gold standard” in practice.
Reference method	A standardised, well-characterised method used for calibration or model development.	Standardised DXA protocol, dilution techniques	Used to calibrate or train BIA models.	Formal term in metrology; implies well-characterised uncertainty and, where feasible, traceability to SI units.
Index method	The technique being tested or evaluated in a study.	SF-BIA, MF-BIA, BIS	Not used to evaluate BIA, since this is the BIA method.	Commonly called the “index test” in diagnostic accuracy studies.

This table clarifies terminology commonly used in body composition method comparison and validation, specifically the terms “gold standard”, “criterion method”, “reference method”, and “index method”, to support consistent interpretation of study results and discussions of BIA in both clinical and metrological contexts. Terminology is used in accordance with principles from the International Vocabulary of Metrology (VIM) [17] and the Guide to the Expression of Uncertainty in Measurement (GUM, JCGM 100:2008) [27]; “gold standard” is retained to reflect common usage in the clinical body-composition literature, although it is not a formal metrological term. * A highly accurate body composition (conceptual) model typically dividing the body into fat, water, bone mineral, and residual mass, using multiple methods (typically DXA, densitometry, and isotope dilution) to minimise error and reduce assumptions [50,98,99].

**Table 9:** Major contributors to uncertainty in BIA-derived body composition estimates.

Source of Uncertainty	Typical Effect	Type	Mechanism	Clinical Implication
Biological variation and hydration status	TBW variability approximately 0.0–0.5 L day-to-day under controlled conditions; substantially larger variability expected in clinical populations [1,44,47,104]	Random/Systematic	Physiological fluctuations in hydration, extracellular/intracellular fluid distribution, inflammation, edema, fasting status, and disease state	May obscure true longitudinal changes and reduce comparability between serial measurements
Body posture and fluid redistribution	Changes in impedance and TBW estimates after transition from standing to supine position; progressive fluid shifts during recumbency may alter impedance by several percent [1,44,104]	Random/Systematic	Gravitational redistribution of extracellular fluid alters limb impedance and conductive pathways	Lack of posture standardization may lead to clinically relevant differences between measurements
Electrode placement	Electrode positioning mismatch may introduce errors of up to approximately 4% in resistance measurements [105,106]	Random/Systematic	Altered current pathways and voltage sensing due to inconsistent anatomical positioning or poor skin-electrode contact	Reduced repeatability and potential systematic bias in derived body composition estimates
Prediction equations and modelling assumptions	SEE for FFM commonly approximately 2–3 kg relative to reference methods; body fat bias approximately −3 to −4% reported for MF-BIA versus DXA [1,3,7,47]	Systematic	Population-specific empirical equations, hydration assumptions, body geometry assumptions, and proprietary algorithms	Major source of systematic bias and limited interchangeability between devices and populations
Device and algorithm differences	Inter-device differences commonly reported despite high within-device repeatability; systematic offsets between technologies may occur [107,108,109,110]	Systematic	Differences in hardware, frequencies, calibration procedures, equivalent circuit models, and embedded algorithms	Measurements from different devices should not automatically be considered interchangeable
Technical repeatability (within-device precision)	Same-day variability approximately 0.0–0.2 L TBW under controlled conditions; ICC values ≥0.999 reported for BIA devices [3,47]	Random	Intrinsic electronic noise and short-term measurement repeatability limitations	Modern BIA devices may demonstrate excellent precision despite remaining clinically relevant systematic uncertainty

This table presents examples of major physiological, methodological, and device-related factors that may influence the accuracy, precision, comparability, and longitudinal interpretation of BIA-derived body composition estimates.

**Table 10:** Common sources of error in BIA and best practice recommendations.

Category	Source of Error	Impact	Recommendations for Best Practices
Patient preparation	Recent food intake, alcohol consumption, or physical exercise.	Alters fluid balance, leading to biased impedance values.	Ensure a fasting period of 4–8 h before measurement, use a consistent time of day, ensure voiding, and allow 5–10 min of rest prior.
Electrode placement	Inconsistent anatomical placement of electrodes.	Changes current paths, which reduces measurement repeatability.	Standardise patient posture (supine or standing), use defined anatomical electrode sites, and ensure clean, dry skin with consistent site marking.
Device calibration	Instrument drift or use of outdated hardware and software.	Introduces systematic errors and variability between devices.	Calibrate devices with known resistors or phantoms, replace worn parts, update firmware, and periodically cross-check multiple devices.
Equation mismatch	Use of incorrect or non-specific prediction equations.	Produces biased body composition estimates, such as underestimation in individuals with obesity.	Select appropriate equations tailored to the population (e.g., healthy, athletic, disease-specific) and validate output against a reference method.
Operator variability	Variation in procedures or techniques between different users.	Results in inconsistent measurement outcomes across different users or procedures.	Implement standard operating procedures (SOPs) and provide structured staff training with competency checks.
Data interpretation	Overinterpretation of small changes in prediction parameters, e.g., FFM and TBW, or raw values, such as R and X_C_.	Increases the risk of misinterpreting the clinical significance of small or isolated values.	Confirm outliers with repeat measurements, avoid overinterpreting small changes that fall within device error, and continuously integrate clinical context.
Documentation and reporting	Incomplete documentation or methodological reporting.	Reduces transparency, reproducibility, comparability, and interpretation across studies and clinical settings.	Document procedural deviations (e.g., recent fluid intake), report raw impedance and derived values, and include device model, software/firmware version, frequency range, electrode configuration, body position, calibration status, prediction equations, population applicability, and repeatability/uncertainty data where available.

This table summarises common BIA error sources, their impact, and strategies to minimise them, providing practical guidance to improve measurement reliability and clinical utility.

## Data Availability

All data are presented in this paper.

## Abbreviations

The following abbreviations are used in this manuscript:

BIA	Bioelectrical impedance analysis
BIS	Bioimpedance spectroscopy
SF-BIA	Single-frequency bioelectrical impedance analysis
MF-BIA	Multi-frequency bioelectrical impedance analysis
BIVA	Bioelectrical impedance vector analysis
DXA	Dual-energy X-ray absorptiometry
TBW	Total body water
ECW	Extracellular water
ICW	Intracellular water
FFM	Fat-free mass
FM	Fat mass
PhA	Phase angle
R	Resistance
X_C_	Capacitive reactance
Z	Impedance
LoA	Limits of agreement
SEE	Standard error of estimate
CV	Coefficient of variation
R_0_ (or R_E_)	Resistance of the extracellular water, measured at zero frequency (*f* = 0)
R_∞_ (or R_INF_)	Resistance at infinite frequency (*f* = ∞)
R_I_	Resistance of the intracellular water
C_m_	Cell membrane capacitance
